# Relationships of wild and domesticated rices (*Oryza* AA genome species) based upon whole chloroplast genome sequences

**DOI:** 10.1038/srep13957

**Published:** 2015-09-10

**Authors:** Peterson W. Wambugu, Marta Brozynska, Agnelo Furtado, Daniel L. Waters, Robert J. Henry

**Affiliations:** 1 Queensland Alliance for Agriculture and Food Innovation, The University of Queensland, Brisbane, St Lucia, Qld, 4072, Australia; 2 Southern Cross Plant Science, Southern Cross University, Lismore, NSW, Australia

## Abstract

Rice is the most important crop in the world, acting as the staple food for over half of the world’s population. The evolutionary relationships of cultivated rice and its wild relatives have remained contentious and inconclusive. Here we report on the use of whole chloroplast sequences to elucidate the evolutionary and phylogenetic relationships in the AA genome *Oryza* species, representing the primary gene pool of rice. This is the first study that has produced a well resolved and strongly supported phylogeny of the AA genome species. The pan tropical distribution of these rice relatives was found to be explained by long distance dispersal within the last million years. The analysis resulted in a clustering pattern that showed strong geographical differentiation. The species were defined in two primary clades with a South American/African clade with two species, *O glumaepatula and O longistaminata*, distinguished from all other species. The largest clade was comprised of an Australian clade including newly identified taxa and the African and Asian clades. This refined knowledge of the relationships between cultivated rice and the related wild species provides a strong foundation for more targeted use of wild genetic resources in rice improvement and efforts to ensure their conservation.

The *Oryza* genus has two cultivated species and about 21 wild relatives and based on chromosome paring, these *Oryza* species are divided into 10 genome types namely AA, BB, CC, BBCC, EE, FF, GG, CCDD, HHJJ and HHKK^1^. The AA genome has eight diploid species among them one of the cultivated species, *O. sativa* L. which has two subspecies, *O. sativa* L. ssp. *japonica and O. sativa* L. ssp. *indica* (hereafter referred as *japonica and indica respectively*) which have a global distribution. The other cultivated species is *O. glaberrima* Steud., commonly referred to as African rice, which is localized in West Africa. These cultivated and wild species hold valuable genetic diversity that has continued to contribute immensely to rice crop improvement. In order to ensure effective conservation and utilization of these resources, knowledge of their evolutionary and phylogenetic relationships is important. The extensive pan-tropical distribution of the AA genome species has long remained an unresolved issue. Theories of Gondwanaland origin and its subsequent breakup as well as long distance dispersal have been used to explain the distribution of *Oryza* species^2^. As reviewed by Wambugu *et al.*^3^, (and the references therein), the persistent incongruence and inconsistency that has characterized efforts to resolve these issues might be attributed to a relatively rapid diversification of the AA genome, differential choice of genes, use of insufficient data, incomplete lineage sorting, misidentification of accessions and introgression.

Previously, phylogenetic analysis^1^,^4^,^5^ has followed the laborious process of amplifying selected loci, some of which unfortunately have not provided sufficient phylogenetic resolution. Recent advances in next generation sequencing have led to the sequencing of various chloroplast genomes which have continued to find utility in various areas of plant science. The use of whole chloroplast genomes, whose potential as a universal barcode has recently been demonstrated^6^, helps to overcome the previously laborious process of data generation. While nuclear data may lead to inconsistent phylogenies due to recombination, plastids have particular advantages in phylogenetic reconstruction as they are structurally stable, generally uniparental, haploid and non-recombinant^7^. Therefore, the aim of this study was to use massively parallel sequencing to reconstruct the evolutionary and phylogenetic relationships of the *Oryza* AA genome species from whole chloroplast genome sequences.

Assembly of paired end reads resulted in nine chloroplast genomes whose sizes ranged from 134563 to 134911 bp with *O. longistaminata* having the smallest genome (Table 1). The assembled genomes had a quadripartite structure which is typical of angiosperms (Fig. 1 and Supplementary results online). Aligning of full chloroplast sequences resulted in an alignment that was 143331bb in length with 134353 characters being constant. A total of 484 characters were variable out of which 221 were parsimony informative (see Supplementary Table S2 online). We obtained a well resolved and strongly supported phylogeny with a strong hierarchy of clades (Fig. 2). The resultant phylogenetic tree had two main distinct clades where the primary division was into a clade including *O. glumaepatula* and *O. longistaminata* which formed the basal clade and one containing all the other species. The large clade in turn contained two well resolved clades, the Australian clade and another one with Asian and African species. The various regions/continents which are used to refer to the different taxa in this study represent their provenance, and in all cases were also the source of collection.

The different phylogenetic criteria namely maximum likelihood, maximum parsimony and neighbour joining produced trees that were congruent, showing the robustness of this approach. There are varied and sometimes contradictory reports on the effect of indels on phylogenetic analysis, with different recommendations being advanced on how they should be treated^8^,^9^. In our study, deletion of indels as is sometimes recommended, resulted in generally poorly resolved and inconsistent phylogeny. Neighbour joining analysis yielded a poorly resolved tree while maximum parsimony and maximum likelihood analysis resulted in a tree that is almost similar to the one we have obtained without deleting the indels, except that *O. glaberrima 2* moved next to the basal clade. Overall, our study shows that deleting the indels prior to phylogenetic analysis may not be an effective approach. Our results are consistent with those of Dessimoz and Gil^10^ who concluded that indels carry substantial phylogenetic signal and deleting them can be detrimental in phylogenetic analysis. Our alignment had long indels and perhaps it is the size of the indels that plays a role in determining their phylogenetic effect.

Among the inconsistencies observed in previous studies, the issue of the radiation of AA genome, basal taxa and divergence time have perhaps been the most outstanding. This study shows that the basal clade consists of *O. longistaminata* and *O. glumaepatula* which are found in Africa and South America respectively. This divergence could be as a result of long distance dispersal through movement of animals especially birds, followed by ecological differentiation. These species are too close to have a divergence due to a pan-Gondwanaland distribution, and the continental drift between Africa and South America which is estimated to have occurred approximately 100–120 Mya^11^,^12^. These two species show a genetic relationship that is common in many other species of African and South American origin^13^,^14^. The presence of the most primitive extant grasses in South America^15^ may also seem to suggest the *Oryza* genus may have had some ancestry here. Molecular divergence dating was conducted using both relaxed and strict clock approaches. The results obtained using the strict clock approach were generally closer to the estimates reported in previous studies, as compared to those obtained using the relaxed clock approach which were generally much older. Strict clock is known to be superior to relaxed clock in case of shallow phylogenies^16^. With the *Oryza* AA genome species having diverged only recently, we believe the strict clock approach provides better divergence date estimates and hence only these estimates will be highlighted in this paper. Based on whole chloroplast genome sequences, this study estimates that the AA genome may have diverged about 0.69–1.11million years ago (Mya) (Table 2 and Fig. 3), a period that seems to correspond to the Pleistocene. This estimate is less than the estimates of 2 Mya given in an earlier study based on intron sequences of four nuclear genes^5^. The results also suggest that the AA genome group diverged from other *Oryza* species around 3.36–5.22 Mya. Relatively recent long distance dispersal remains the most reliable explanation for the current distribution of AA genome species.

Though chloroplast genomes may have particular advantages in resolving phylogenetic relationships, Middleton *et al.*^17^, noted that their use in divergence dating may be problematic especially in species with short evolutionary times. This is due to the fact that presence of multiple chloroplast haplotypes may not correspond with species divergence since they may have diverged long before actual species divergence. This may therefore lead to inaccurate divergence estimates and may explain the difference between nuclear based and chloroplast based divergence estimates. Increasing the amount of data analysed in divergence dating has been found to result in more accurate estimates^18^ and we therefore believe that due to the use of a large data set, our estimates are robust and reliable.

The origin of the AA genome has also remained quite contentious with different arguments and opinions being advanced. Previous studies suggest the AA genome group may have originated from Africa since the majority of the studies have reported *O. longistaminata* as the most ancestral species^19^,^20^. Furthermore, *O. longistaminata* has unique morphological features that are not present in other AA genome species such as self-incompatibility, rhizomatous and unique characteristics of the ligules, all consistent with it being significantly differentiated. The conventional wisdom in evolutionary biology is that annual species are derived from perennial ancestors. This is consistent with our findings which indicate that *O. longistaminata* and *O. glumaepatula*, both of which are perennial, are the most ancestral species.

The basal split in this study was followed by the divergence of the Australian species from the Asian and African taxa and is estimated to have occurred about 0.54–0.86 Mya. Contrary to some studies which show that the spread of *Oryza* A genome species into Australia may have occurred earlier^4^, this study shows that spread to all the regions seems to have occurred just around the same time, an observation earlier made by Vaughan *et al.*^21^. The sharing of some taxa such as some types of *Sorghum, Vigna radiata* var. *sublobata* and *Gossypium* species between Australia and Africa suggests that there is a phytogeographic link between the two regions^22^. This study shows that the divergence between the African and Asian species occurred twice, with the first one being the divergence between ancestors of *O. longistaminata* and those of the Asian species while the second one is the radiation of the ancestors of the two cultivated taxa. The divergence between the two cultivated taxa is estimated to have occurred about 0.51–0.65 Mya (Table 2 and Fig. 3). The timing of this divergence corresponds to estimates given in previous studies of 0.64–0.7 Mya^5^,^23^. However, this estimated time of divergence is much earlier than the time of domestication of Asian and African rice, which is reported to have taken place about 10,000 years and 3500 years ago respectively.

Many conflicting theories have been advanced on the origin and domestication of Asian cultivated rice and despite great research efforts, the debate rages on. Traditionally, these theories were broadly classified as supporting either monophyletic^24^,^25^ or polyphyletic origin^26^,^27^. Recently however, some domestication pathways that deviate from these previously suggested models have been proposed^28^. In one of these models, Huang *et al.*^24^, suggested that *japonica* was domesticated from *O. rufipogon* in Southern China while *indica* was the product of a cross between *japonica* and local wild rice in South East Asia and South Asia. Analysis of genetic polymorphisms between the *O. sativa* subspecies shows some genetic differentiation with *indica* 1 and *indica* 2 having 296 and 247 nucleotide differences respectively between them and the japonica reference (GU592207). This genetic differentiation is confirmed by their phylogenetic placement where both *japonica* and *indica* formed distinct clades, associated with *O. rufipogon* and *O. nivara* respectively. This genetic differentiation between the two subspecies of *O. sativa* and the associated clustering pattern seems to support polyphyletic evolution, and suggests independent domestication of Asian rice as opposed to the single origin theory. Proponents of the independent domestication theory, posit that the diverged genomic backgrounds of *japonica* and *indica* were derived from pre-differentiated ancestral gene pools during separate and geographically isolated domestication events. This theory has gained support from several phylogenetic, genetic distance and genomic palaeontology analyses. Our findings suggest the possibility that the maternal genome of *indica* may have been derived from *O. nivara* while the maternal genome of *japonica* may have originated from *O. rufipogon*. The nuclear genomes may have a more complex origin.

Similar to Asian rice, the origin of African rice has also been controversial and remains grossly understudied. Although the widely held proposition is that African rice was domesticated from *O. barthii*^29^,^30^, it has also been hypothesized that it evolved from Asian rice through sympatric speciation before it was later domesticated in West Africa^31^. According to this theory, upon introduction of Asian rice to Africa, drastic changes in climate and especially the high temperatures in the Sahel may have led to mutations which may have subsequently been selected for by farmers. This latter theory has however not gained much support and the arguments presented in its support seem less convincing. An Asian origin of African rice has also been proposed but dismissed^29^. The shattering nature of *O. glaberrima* and presence of red pericarp in some varieties seems to suggest that the process of domestication is incomplete. In the African clade, *O. glaberrima* clustered with all the four accessions of *O. barthii*, thus supporting the popular hypothesis of *O. barthii* being the progenitor of African rice. A recent study resequenced 20 accessions of *O. glaberrima* and 94 of *O. barthii*^30^ and their results supported the domestication theory originally proposed by Portères^32^ which was later supported by Li *et al.*^29^. Portères suggested that African rice was first domesticated in the Inland Delta of the Upper Niger River before spreading to two secondary centres, one located along the Senegambian coast and the other in the Guinea highlands^32^. The wide inconsistencies in evolutionary and phylogenetic relationships may be attributed to the use of germplasm that has been collected from areas that have been disturbed through human activity as well as those that are misidentified. Such human activities may have eroded important genetic signatures thus leading to contradictory theories and conclusions.

The strong geographic differentiation among the “*O. rufipogon”* accessions from either Asia or Australia was evidently clear. Information on such genetic patterns is valuable in guiding both germplasm use and conservation efforts particularly when devising sampling strategies. Though the observed genetic differentiation in “*O. rufipogon”* accessions from different regions is consistent with previous analysis conducted using molecular^33^ and hybridization data^34^ which showed them as distinctly different taxa, it should be interpreted with caution. The clustering of “*O. rufipogon*” in different clades may be due to incomplete lineage sorting or introgressive hybridization which may have led to chloroplast capture^35^,^36^. *O. rufipogon* is an outcrossing species^34^ and so chloroplast capture is possible especially with species with which it has sympatric distribution^37^. Introgression is more frequent in chloroplast than in nuclear genomes and this phenomenon of reticulation and chloroplast capture usually leads to incongruence between nuclear and chloroplast based phylogenies^35^. It has generally been demonstrated that chloroplast based phylogenies usually reflect geographical distribution as compared to nuclear phylogenies which normally correspond with taxonomic relationships^37^,^38^,^39^. As recommended by Rieseberg and Soltis^35^, conducting phylogenetic analysis using both nuclear and chloroplast genomes is an important strategy to avoid erroneous phylogenies. The use of unlinked nuclear genes would be useful in testing for incomplete lineage sorting and this study therefore serves as a useful starting point, whose findings can be compared with the analysis of selected nuclear loci.

Recent studies using both molecular and morphological data have discovered two distinct perennial taxa in Northern Queensland, Australia which are believed to be new *Oryza* gene pools^40^,^41^. One of these taxa has previously been identified as *O. rufipogon*^40^. With the chloroplast genome of one of the two perennial taxa closely resembling that of the annual *O. meridionalis*^33^, there is need for further work to assess the possibility of introgressive hybridization resulting in chloroplast capture as already discussed above. The discovery of these gene pools has a great impact on global food security as it provides the breeders with critical genetic resources. The *Oryza* gene pools in South America/Africa and Australia are likely to possess important traits as they are genetically isolated from domesticated rice and hence remain genetically uncontaminated by gene flow from the large Asian domesticated rice populations.

## Methods

### Plant materials

The seed samples of African *Oryza* wild species used in this study were obtained from the International rice research institute (IRRI), Philippines while those of *O. glumaepatula* and *O. officinalis* were obtained from the Queensland Alliance for Agriculture and Food Innovation (QAAFI), Australia (see Supplementary Table S1 online).

### DNA extraction and sequence assembly

DNA was extracted from approximately 3 gm of plant tissue using a modified cetyltrimethylammonium bromide (CTAB) method that was originally published by Carroll, *et al.*^42^ The DNA quality and quantity was assessed by agarose gel electrophoresis (0.7%, 103 V for 45 minutes) as well as by an Agilent BioAnalyzer 2100 (Agilent Technologies). Approximately 3 μg of total DNA from each accession was indexed and pooled together in one lane of an Illumina Genome Analyzer and sequenced. In total, we sequenced 9 *Oryza* chloroplast genomes representing five species. The raw reads were assembled into whole chloroplast genomes in a multi-step approach employing a modified pipeline that involved a combination of both reference guided and *de novo* assembly approaches^43^. First, trimming was done with a quality score limit of 0.05 on CLC Genomics workbench 6.5.1 before undertaking sequence assembly. In order to discard non-CP reads, the trimmed reads were assembled by mapping to the published *O. sativa* chloroplast reference sequence (GU592207) using CLC Genomic Workbench 6.5.1 under the following parameters: length fraction 0.5, similarity fraction 0.8, mismatch cost 2, deletion and insertion costs 3. In order to allow assembly of the inverted repeats as well as other repetitive elements, reads showing non-specific matches were mapped randomly. The vote majority conflict resolution mode was used in order to ensure inclusion of only chloroplast specific reads thus avoiding contribution of nuclear and mitochondria reads to the consensus sequences. With the aim of correcting any assembly errors especially relating to possible misalignment due to insertions and deletions, the read mapping consensus sequences obtained were subjected to two iterations of forced realignment using the respective InDel variant tracks.

After reference guided assembly, *de novo* assembly was undertaken with a minimum contig length of 1000 bp. The resultant *de novo* contigs were aligned to the reference sequence using BLAST (http://blast.ncbi.nlm.nih.gov/) as implemented in CLC Genomic Workbench 6.5.1 and the matching cp contigs ordered by alignment to the reference sequence using clone manager 9.0. The trimmed reads were later mapped to the *de novo* assembled sequence as reference and the resultant consensus sequence subjected to one cycle of forced realignment using InDel guidance variant tracks. The *de novo* and reference guided consensus were then compared and any conflicts resolved by reference to the reads.

### Phylogenetic analysis

The final chloroplast sequences from *O. barthii, O. longistaminata, O. glaberrima, O. glumaepatula* and *O. officinalis* were exported to Geneious 7.0.5 (www.geneious.com) where, using MAFFT^44^, they were aligned with chloroplast sequences for O. *meridionalis*, *O. nivara, O. rufipogon, japonica and indica* which were downloaded from Genbank (see Supplementary Table S3 online). The alignment was physically inspected and confirmed to be correct. The number of variable characters and parsimony informative sites were analysed in MEGA6 (ref. 45) with the out-group excluded. The aligned sequences were analysed by maximum parsimony (MP), neighbour joining (NJ), maximum likelihood (ML) and Bayesian Inference criteria using PAUP 4.0b10 (ref. 46) implemented through Geneious v 7.0.5. The Neighbour joining criterion used the general time-reversible (GTR) distance measure with rates assumed to follow gamma distribution. The maximum parsimony analysis was performed using heuristic searches with the ‘MulTrees’ option followed by tree bisection–reconnection branch swapping. Topological robustness was assessed using bootstrap method with 1000 random addition replicates. All characters were unordered and were accorded equal weight with gaps being treated as missing data. A separate phylogenetic assessment was also conducted with the gaps being deleted manually but resulted in a poorly resolved and inconsistent tree. Appropriate nucleotide substitution models were determined using Modeltest 3.7 (ref. 47). The models were chosen using Akaike Information Criterion (AIC) with the models being used for subsequent maximum likelihood and Bayesian Inference analysis. Maximum likelihood analysis was undertaken using the GTR+G+I model. Bayesian phylogenetic inference was conducted using MrBayes 3.1 (ref. 48) with Monte Carlo Markov Chains (MCMC) estimation of posterior probability distributions. The analysis used the GTR model with the rate variation assumed to follow gamma distribution. Four independent runs of 1,100,000 MCMC were performed with trees sampled after every 200 runs followed by a burn in length of 100,000 MCMC. *O. officinalis* acted as the out group.

### Molecular divergence dating

Molecular clock analysis was conducted using Bayesian method implemented in BEAST program^49^. The analysis was conducted using both strict and relaxed clock approaches with the data being partitioned into coding, non-coding and intergenic sequences. Calibration was done based on the divergence time between *Zea mays* and *Triticum aestivum* which is estimated to have occurred about 50 Mya^50^. The HKY and four gamma categories substitution model was used with the Calibrated Yule model priors being used to model speciation. TreeAnnotator (pre-release version 2.1.2) was used to calculate maximum probability clade tree and the final tree was visualized in FigTree v1.4.2.

### Genome annotation

Annotation of the sequenced genomes was done using CpGAVAS^51^ (http://www.herbalgenomics.org/0506/cpgavas) followed by manual adjustments of start and stop codons as well as intron/exon boundaries which was done using Apollo^52^. GenBank files were created using sequin and were subsequently used to draw chloroplast gene maps using OGDraw v1.2 (ref. 53).

## Additional Information

**Accession codes**: The assembled complete chloroplast genomes have been deposited under GenBank accession numbers KM881635- KM881643.

**How to cite this article**: Wambugu, P. W. *et al.* Relationships of wild and domesticated rices (*Oryza* AA genome species) based upon whole chloroplast genome sequences. *Sci. Rep.* **5**, 13957; doi: 10.1038/srep13957 (2015).

## Supplementary Material

Supplementary Information

## Figures and Tables

**Figure 1:**
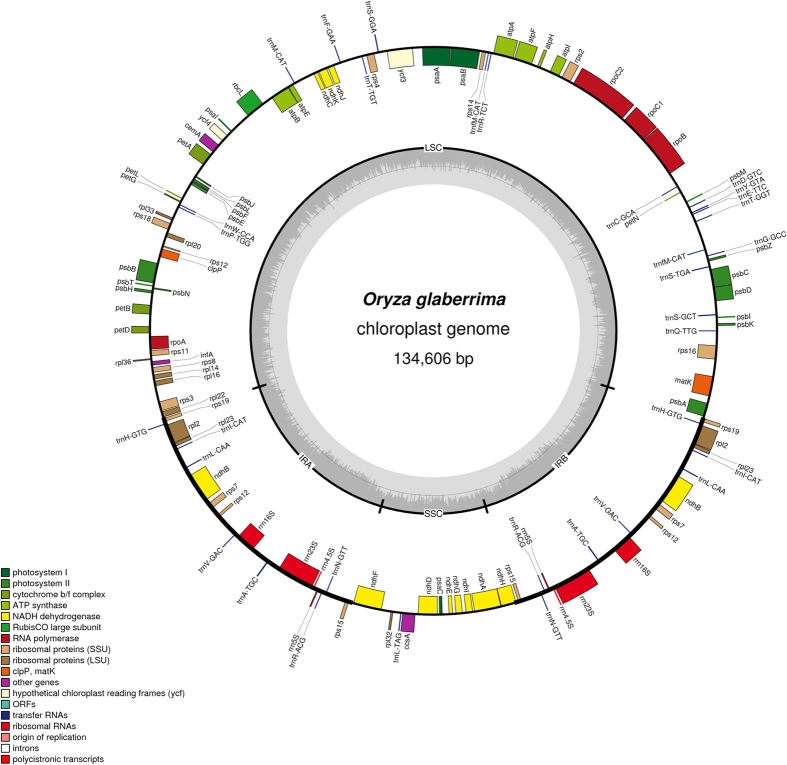
Gene map of *O. glaberrima* chloroplast genome. The different types of genes are colour coded. Genes shown inside are transcribed clockwise while those on the outside are transcribed anticlockwise. The inner circle represents the two inverted repeats (IRA and IRB) which are separated by the short single copy (SSC) and the long single copy (LSC).

**Figure 2:**
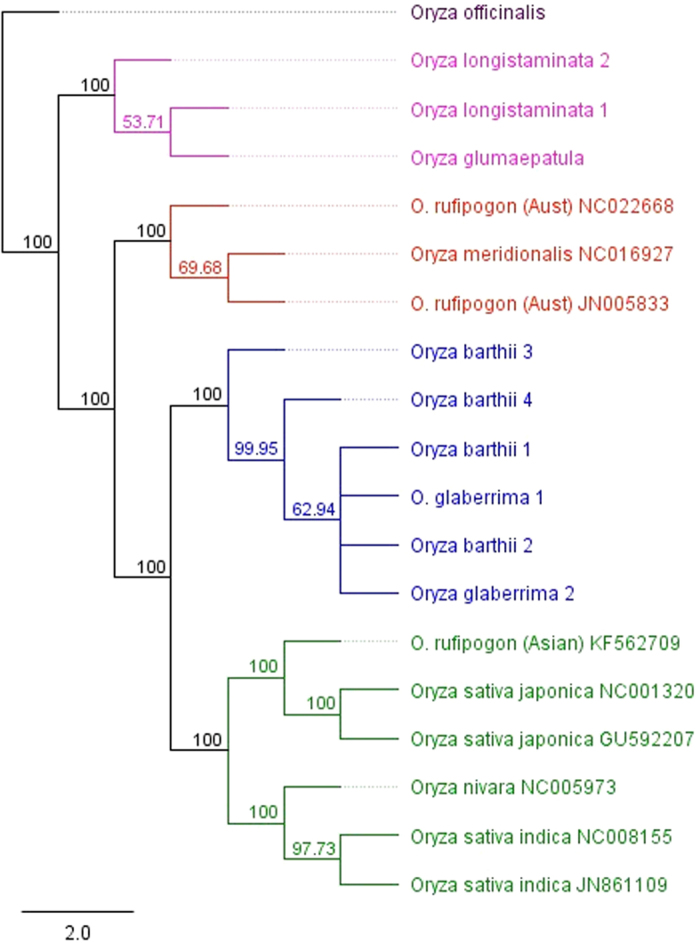
Bootstrapping consensus and maximum parsimony tree showing phylogenetic relationships among *Oryza* AA genome species conducted using whole chloroplast sequences. *O. officinalis* acted as the out-group. The same topology was obtained from Neighbour joining and maximum likelihood criteria. Indicated accession numbers are GenBank unique identifiers. The same topology was obtained when one of the inverted repeat regions was excluded. Deleting the indels resulted in a poorly resolved and inconsistent phylogeny.

**Figure 3:**
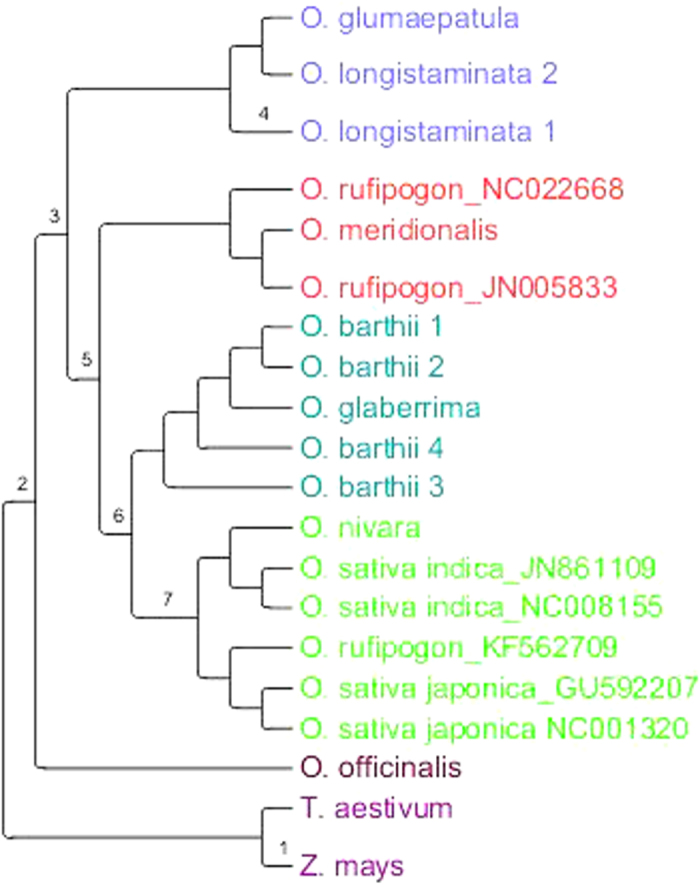
Phylogenetic tree showing relationships among *Oryza* AA genome species based on whole chloroplast sequences. The divergence dates for the various labelled nodes were calculated using strict and relaxed clocks and are shown in Table 2. Divergence between *Triticum aestivum* and *Zea mays* was used as the calibration point while *O. officinalis* acted as the out-group. The tree is not drawn to scale.

**Table 1:** Summary statistics of the chloroplast genome sequencing and assembly.

Species	Total paired-end reads	Average length (after trimming)	No. of Reads aligning to reference	Proportion of reads aligning to reference (%)	Fold coverage	Cp genome size	Accession number
*O. longistaminata 1*	38275022	96.8	4786403	12.6	3443	134567	KM881641
*O. longistaminata 2*	38613960	96.7	4398194	11.5	3160	134563	KM881642
*O. barthii 1*	34562744	96.5	3387828	9.92	2427	134674	KM881634
*O. barthii 2*	35661888	96.5	3574521	10.12	2562	134603	KM881635
*O. barthii 3*	35336280	96.5	2304561	6.58	1652	134596	KM881636
*O. barthii 4*	35095334	96.4	2205372	6.35	1579	134640	KM881637
*O. glaberrima 1*	35220340	96.8	3695118	10.68	2657	134606	KM881638
*O. glumaepatula*	36220444	96.6	1838613	5.13	1319	134583	KM881640
*O. officinalis*	38710520	96.7	1935941	5.1	1387	134911	KM881643

**Table 2:** Divergence date estimates of the AA *Oryza* genome species based on chloroplast sequences.

Node label^a^	Divergence date estimates^b^	
	Strict clock^c^	Relaxed clock
1	48.93 (39.7–58.9)	49.41(49.21–50.99)
2	4.37 (3.36–5.22)	41.76 (34.94–51.48)
3	0.95 (0.69–1.11)	17.23 (8.49–35.55)
4	0.2 (0.1–0.23)	5.32 (1.27–10.87)
5	0.86 (0.61–0.97)	11.99 (7.41–19.82)
6	0.77 (0.54–0.86)	7.62 (2.68–12.73)
7	0.68 (0.5–0.78)	6.66 (2.95–11.62)

^a^Nodes are labelled in the phylogenetic tree shown in Fig. 3.

^b^Divergence estimates are in millions of years.

^c^95% HPD (Highest Posterior Density) is indicated in parenthesis.

## References

[b1] Ge S., Sang T., Lu B.-R. & Hong D.-Y. Phylogeny of rice genomes with emphasis on origins of allotetraploid species. Proc. Natl. Acad. Sci. USA 96, 14400–14405 (1999). 10588717 10.1073/pnas.96.25.14400 PMC24448

[b2] Chang T.-T. The origin, evolution, cultivation, dissemination, and diversification of Asian and African rices. Euphytica 25, 425–441 (1976).

[b3] Wambugu P., Furtado A., Waters D., Nyamongo D. & Henry R. Conservation and utilization of African *Oryza* genetic resources. Rice 6, 29 (2013). 24280189 10.1186/1939-8433-6-29 PMC4883696

[b4] Zhu T. *et al.* Phylogenetic relationships and genome divergence among the AA- genome species of the genus *Oryza* as revealed by 53 nuclear genes and 16 intergenic regions. Mol. Phylogenet. Evol. 70, 348–361 (2014). 24148990 10.1016/j.ympev.2013.10.008

[b5] Zhu Q. & Ge S. Phylogenetic relationships among A-genome species of the genus *Oryza* revealed by intron sequences of four nuclear genes. New Phytol. 167, 249–265 (2005). 15948847 10.1111/j.1469-8137.2005.01406.x

[b6] Nock C. J. *et al.* Chloroplast genome sequences from total DNA for plant identification. Plant Biotechnol. J. 9, 328–333 (2011). 20796245 10.1111/j.1467-7652.2010.00558.x

[b7] Small R. L., Cronn R. C. & Wendel J. F. Use of nuclear genes for phylogeny reconstruction in plants. Aust. Syst. Bot. 17, 145–170 (2004).

[b8] Nagy L. G. *et al.* Re-Mind the Gap! Insertion - Deletion Data Reveal Neglected Phylogenetic Potential of the Nuclear Ribosomal Internal Transcribed Spacer (ITS) of Fungi. PloS one 7, e49794 (2012). 23185439 10.1371/journal.pone.0049794 PMC3501463

[b9] Ogden T. H. & Rosenberg M. S. How should gaps be treated in parsimony? A comparison of approaches using simulation. Mol. Phylogenet. Evol. 42, 817–826 (2007). 17011794 10.1016/j.ympev.2006.07.021

[b10] Dessimoz C. & Gil M. Phylogenetic assessment of alignments reveals neglected tree signal in gaps. Genome Biol. 11, R37–R37 (2010). 20370897 10.1186/gb-2010-11-4-r37 PMC2884540

[b11] Wood A. E. [The History of the Problem] The Africa-South America Connection [Wlma G. & Rene L. (eds)] [1–7] (Oxford university press, Oxford, 1993).

[b12] Duncan R. A. & Hargraves R. B. [Plate tectonic evolution of the Caribbean region in the mantle reference frame] *The Caribbean- South American Plate Boundary and Regional Tectonics* Vol. Memoir 162 [Bonini W. E. & Hargraves R. S. (eds)] [81–95] (Geological Society of America, 1984).

[b13] Groeneveld L. F., Clausnitzer V. & Hadrys H. Convergent evolution of gigantism in damselflies of Africa and South America? Evidence from nuclear and mitochondrial sequence data. Mol. Phylogenet. Evol. 42, 339–346 (2007). 16945555 10.1016/j.ympev.2006.05.040

[b14] Givnish T. J. *et al.* Ancient vicariance or recent long-distance dispersal? Inferences about phylogeny and South American-African disjunctions in Rapateaceae and Bromeliaceae based on *ndhF* sequence data. Int. J. Plant Sci. 165, S35–S54 (2004).

[b15] Bremer K. Early Cretaceous lineages of monocot flowering plants. Proc. Natl. Acad. Sci. USA 97, 4707–4711 (2000). 10759567 10.1073/pnas.080421597 PMC18297

[b16] Brown R. P. & Yang Z. Rate variation and estimation of divergence times using strict and relaxed clocks. BMC Evol. Biol. 11, 271–271 (2011). 21943087 10.1186/1471-2148-11-271 PMC3205074

[b17] Middleton C. P. *et al.* Sequencing of chloroplast genomes from wheat, barley, rye and their relatives provides a detailed insight into the evolution of the Triticeae tribe. PloS One 9, e85761 (2014). 24614886 10.1371/journal.pone.0085761 PMC3948623

[b18] Kumar S. & Hedges S. B. A molecular timescale for vertebrate evolution. Nature 392, 917–920 (1998). 9582070 10.1038/31927

[b19] Iwamoto M., Nagashima H., Nagamine T., Higo H. & Higo K. p-SINE1-like intron of the CatA catalase homologs and phylogenetic relationships among AA-genome *Oryza* and related species. Theor. Appl. Genet. 98, 853–861 (1999).

[b20] Cheng C., Tsuchimoto S., Ohtsubo H. & Ohtsubo E. Evolutionary relationships among rice species with AA genome based on SINE insertion analysis. Genes Genet. Syst. 77, 323–334 (2002). 12441643 10.1266/ggs.77.323

[b21] Vaughan D. A., Kadowaki K.-i., Kaga A. & Tomooka N. On the phylogeny and biogeography of the genus *Oryza*. Breeding Sci. 55, 113–122 (2005).

[b22] Vaughan D. A., Ge S., Kaga A. & Tomooka N. [Phylogeny and Biogeography of the Genus *Oryza*] *Rice Biology in the Genomics Era* [Hirano H.-Y., Hirai A., Sano Y. & Sasaki T. (eds)] [219–234] (Springer, Berlin Heidelberg, 2008).

[b23] Ma J. & Bennetzen J. L. Rapid recent growth and divergence of rice nuclear genomes. Proc. Natl. Acad. Sci. USA 101, 12404–12410 (2004). 15240870 10.1073/pnas.0403715101 PMC515075

[b24] Molina J. *et al.* Molecular evidence for a single evolutionary origin of domesticated rice. Proc. Natl. Acad. Sci. USA 108, 8351–8356 (2011). 21536870 10.1073/pnas.1104686108 PMC3101000

[b25] Joshi S. P., Gupta V. S., Aggarwal R. K., Ranjekar P. K. & Brar D. S. Genetic diversity and phylogenetic relationship as revealed by inter simple sequence repeat (ISSR) polymorphism in the genus *Oryza*. Theor. Appl. Genet. 100, 1311–1320 (2000).

[b26] Bautista N. S., Solis R., Kamijima O. & Ishii T. RAPD, RFLP and SSLP analyses of phylogenetic relationships between cultivated and wild species of rice. Genes Genet. Syst. 76, 71–79 (2001). 11434461 10.1266/ggs.76.71

[b27] Kumagai M., Wang L. & Ueda S. Genetic diversity and evolutionary relationships in genus *Oryza* revealed by using highly variable regions of chloroplast DNA. Gene 462, 44–51 (2010). 20450965 10.1016/j.gene.2010.04.013

[b28] Huang X. *et al.* A map of rice genome variation reveals the origin of cultivated rice. Nature 490, 497–501 (2012). 23034647 10.1038/nature11532 PMC7518720

[b29] Li Z. M., Zheng X. M. & Ge S. Genetic diversity and domestication history of African rice (*Oryza glaberrima*) as inferred from multiple gene sequences. Theor. Appl. Genet. 123, 21–31 (2011). 21400109 10.1007/s00122-011-1563-2

[b30] Wang M. H. *et al.* The genome sequence of African rice (*Oryza glaberrima*) and evidence for independent domestication. Nat. Genet. 46, 982–988 (2014). 25064006 10.1038/ng.3044 PMC7036042

[b31] Nayar N. M. [The Origin of African Rice] *Origins and Phylogeny of Rices [117-168]* (Elsevier Science, Burlington, 2014).

[b32] Porteres R. Berceaux Agricoles Primaires Sur le Continent Africain. J. Afr. Hist. 3, 195–210 (1962).

[b33] Waters D. L. E., Nock C. J., Ishikawa R., Rice N. & Henry R. J. Chloroplast genome sequence confirms distinctness of Australian and Asian wild rice. Eco. Evol. 2, 211–217 (2012). 10.1002/ece3.66 PMC3297189 22408737

[b34] Banaticla-Hilario M. C. N., McNally K. L., Berg R. G. & Hamilton N. R. S. Crossability patterns within and among *Oryza* series Sativae species from Asia and Australia. Genet. Resour. Crop Evol. 60, 1899–1914 (2013).

[b35] Rieseberg L. H. & Soltis D. E. Phylogenetic consequences of cytoplasmic gene flow in plants. Evol. Trend Plants 5, 65–84 (1991).

[b36] Zou X. H. *et al.* Analysis of 142 genes resolves the rapid diversification of the rice genus. Genome Biol. 9, R49–R49 (2008). 18315873 10.1186/gb-2008-9-3-r49 PMC2397501

[b37] Cristina Acosta M. & Premoli A. C. Evidence of chloroplast capture in South American Nothofagus (subgenus Nothofagus, Nothofagaceae). Mol. Phylogenet. Evol. 54, 235–242 (2010). 19683588 10.1016/j.ympev.2009.08.008

[b38] Rautenberg A. *et al.* Geographic and phylogenetic patterns in Silene section Melandrium (Caryophyllaceae) as inferred from chloroplast and nuclear DNA sequences. Mol. Phylogenet. Evol. 57, 978–991 (2010). 20723610 10.1016/j.ympev.2010.08.003

[b39] Wysocki W. P., Clark L. G., Attigala L., Ruiz-Sanchez E. & Duvall M. R. Evolution of the bamboos (Bambusoideae; Poaceae): a full plastome phylogenomic analysis. BMC Evol. Biol. 15, 50 (2015). 25887467 10.1186/s12862-015-0321-5 PMC4389303

[b40] Brozynska M. *et al.* Chloroplast genome of novel rice germplasm identified in Northern Australia. Trop. Plant Biol. 7, 111–120 (2014). 25485030 10.1007/s12042-014-9142-8 PMC4245483

[b41] Sotowa M. *et al.* Molecular relationships between Australian annual wild rice, *Oryza meridionalis*, and two related perennial forms. Rice 6, 26 (2013). 24280095 10.1186/1939-8433-6-26 PMC3874672

[b42] Carroll B. J. *et al.* Germinal transpositions of the maize element Dissociation from T-DNA loci in tomato. Genetics 139, 407–420 (1995). 7705641 10.1093/genetics/139.1.407 PMC1206337

[b43] Cronn R. *et al.* Multiplex sequencing of plant chloroplast genomes using Solexa sequencing-by-synthesis technology. Nucleic Acids Res. 36, e122–e122 (2008). 18753151 10.1093/nar/gkn502 PMC2577356

[b44] Katoh K., Misawa K., Kuma K.-i. & Miyata T. MAFFT: a novel method for rapid multiple sequence alignment based on fast Fourier transform. Nucleic Acids Res. 30, 3059–3066 (2002). 12136088 10.1093/nar/gkf436 PMC135756

[b45] Tamura K., Stecher G., Peterson D., Filipski A. & Kumar S. MEGA6: Molecular Evolutionary Genetics Analysis version 6.0. Mol. Biol. Evol. 30, 2725–2729 (2013). 24132122 10.1093/molbev/mst197 PMC3840312

[b46] Swofford D. L. PAUP*. Phylogenetic Analysis Using Parsimony (*and Other Methods). Version 4 (Sinauer Associates, Sunderland, MA, 2002).

[b47] Posada D. & Crandall K. A. MODELTEST: testing the model of DNA substitution. Bioinformatics 14, 817–818 (1998). 9918953 10.1093/bioinformatics/14.9.817

[b48] Ronquist F. & Huelsenbeck J. P. MrBayes 3: Bayesian phylogenetic inference under mixed models. Bioinformatics 19, 1572–1574 (2003). 12912839 10.1093/bioinformatics/btg180

[b49] Bouckaert R. *et al.* BEAST 2: a software platform for Bayesian evolutionary analysis. PLoS Comput. Biol. 10, e1003537 (2014). 24722319 10.1371/journal.pcbi.1003537 PMC3985171

[b50] Wolfe K. H., Gouy M., Yang Y. W., Sharp P. M. & Li W. H. Date of the monocot-dicot divergence estimated from chloroplast DNA sequence data. Proc. Natl. Acad. Sci. USA 86, 6201–6205 (1989). 2762323 10.1073/pnas.86.16.6201 PMC297805

[b51] Liu C. *et al.* CpGAVAS, an integrated web server for the annotation, visualization, analysis, and GenBank submission of completely sequenced chloroplast genome sequences. BMC Genomics 13, 715–715 (2012). 23256920 10.1186/1471-2164-13-715 PMC3543216

[b52] Lewis S. E. *et al.* Apollo: a sequence annotation editor. Genome Biol. 3, 0082 (2002). 10.1186/gb-2002-3-12-research0082 PMC151184 12537571

[b53] Lohse M., Drechsel O., Kahlau S. & Bock R. OrganellarGenomeDRAW-a suite of tools for generating physical maps of plastid and mitochondrial genomes and visualizing expression data sets. Nucleic Acids Res. 41, W575–W581 (2013). 23609545 10.1093/nar/gkt289 PMC3692101

