# Supplementary material for: Relationships of wild and domesticated rices (Oryza AA genome species) based upon whole chloroplast genome sequences
Source: Sci Rep. 2015 Sep 10;5:13957. doi: 10.1038/srep13957 (PMC4564799; doi:10.1038/srep13957)
Supplement: Supplementary Information [file srep13957-s1.pdf]

Supplementary information

Relationships of wild and domesticated rices (*Oryza* AA genome species) based upon whole chloroplast genome sequences

Peterson W. Wambugu[1], Marta Brozynska[1], Agnelo Furtado[1], Daniel L. Waters[2] and Robert J. Henry[1*]

[1]Queensland Alliance for Agriculture and Food Innovation, The University of Queensland, Brisbane, St Lucia, Qld, 4072, Australia
[2]Southern Cross Plant Science, Southern Cross University, Lismore, NSW, Australia

Supplementary Tables

Supplementary Table S1: Details of seed samples used for DNA extraction

Species	Accession number	Country of origin
Oryza glaberrima	104040	Chad
Oryza longistaminata	103905	Tanzania
Oryza longistaminata	81953	Zambia
Oryza barthii	103590	Cameroon
Oryza barthii	101381	Niger
Oryza barthii	104124	Chad
Oryza barthii	86481	Zambia
Oryza glumaepatula	AusTRCF 309281	Suriname
Oryza officinalis	AusTRCF 309302	Myanmar

Supplementary Table S2: Alignment statistics for whole chloroplast genome and with one inverted repeat (IR) excluded

Statistics/parameter	Whole cp genome	Cp genome with one IR excluded[a]
Alignment length	143,331bp	114,151bp
Conserved sites	134,353	113,591
Variable sites	484	439
Parsimony informative sites	221	214
Singleton sites	262	225
GC content	39.0	38.0

[a] *Oryza glaberrima* 2 was excluded in this analysis as it was not possible to accurately identify the inverted repeats (IR) due to suspected assembly errors.

Supplementary Table S3: GenBank sequences used in this study

Species	Length (bp)	Accession number
O. sativa sp. *Japonica 1*	134,551	GU592207
O. sativa sp. *Japonica 2*	134,525	NC_001320
O. sativa sp. *Indica 1*	134,448	JN861109
O. sativa sp. Indica 2	134,496	NC_008155
O. rufipogon 1	134,537	KF562709
O. rufipogon 2	134,557	NC_022668
O. rufipogon 3	134,,557	JN005833
O. meridionalis	134,558	NC_016927
O. nivara	134,494	NC_005973
O. glaberrima 2	132,629	KJ513090

Supplementary results

Sequencing of 9 genomes produced a varying number of paired reads that ranged from 34562744 - 38275022. Reference guided assembly resulted in mapping rates that ranged from 5.1% to 12.6% which is equivalent to chloroplast genome fold coverage of between 1319X and 3443X (Table 1). The assembled genomes had a quadripartite structure comprising of a long single copy (80512-80684bp) and a short single copy (12336-12386bp) separated by a pair of inverted repeat regions (20794-20809bp) (Fig. 1). Each genome had a total of 124 unique genes comprising of 83 protein coding genes, 33 transfer RNA (tRNA) and 8 ribosomal RNA (rRNA) genes.
